# Reconstruction of the Peroneus Brevis Tendon Tears with Semitendinosus Tendon Autograft

**DOI:** 10.1155/2019/5014687

**Published:** 2019-06-11

**Authors:** Danilo Ryuko Cândido Nishikawa, Fernando Aires Duarte, Guilherme Honda Saito, Cesar de Cesar Netto, Augusto César Monteiro, Marcelo Pires Prado, Ivan Furlan Grava de Sousa

**Affiliations:** ^1^Department of Orthopedics Foot and Ankle Surgery, Hospital do Servidor Municipal de São Paulo (HSPM), 60 Castro Alves Street, Aclimação, CEP: 01532-000 São Paulo, SP, Brazil; ^2^Department of Orthopaedic Surgery, Hospital Israelita Albert Einstein, 627 Albert Einstein Avenue Jardim Leonor, CEP: 05652-900 São Paulo, SP, Brazil; ^3^Department of Orthopedics Foot and Ankle Surgery, Medstar Union Memorial Hospital, 33rd Street Building, 200 E 33rd ST#501, Baltimore, MD, USA

## Abstract

Peroneal tendon disorders are common causes of lateral and retromalleolar ankle pain. For irreparable tears of the tendon, a salvage procedure is indicated with segmental resection followed by reconstruction with tenodesis, tendon transfer, or bridging the defect using allograft or autograft. Although there is insufficient evidence to guide which of these treatment options provides the best outcomes, reconstruction with tendon allograft has provided satisfactory clinical results and is effective for pain relief and restoration of tendon function. However, there are concerns about the use of tendon allografts which include its cost and availability, disease transmission, delayed incorporation, and stretching of the graft. The aim of this study is to present the surgical technique for the reconstruction of the peroneus brevis tendon tears using semitendinosus tendon autograft as an alternative to the allograft and report the short-term results of three cases.

## 1. Introduction

Peroneal tendon (PT) disorders are common causes of lateral and retromalleolar ankle pain [1–3]. PT injuries include tenosynovitis, chronic tendinopathy, subluxation and dislocation, longitudinal splits, partial or complete tears, and painful os peroneum syndrome [4–6]. Chronic conditions and anatomic factors have been implicated as causes of abnormalities such as chronic lateral ankle instability, cavovarus foot, low-lying peroneus brevis (PB) muscle belly, and peroneus quartus tendon [7–10].

The current options of treatment for PT injuries include the following: (1) nonoperative treatment, (2) peroneal tendoscopy, (3) opened debridement and tubularization of the remaining tendon, (4) tenodesis, (5) tendon transfer of the flexor hallucis longus or flexor digitorum longus, and (6) reconstruction with allograft or autograft [6, 11–13]. When the tears are irreparable, a salvage procedure is indicated with segmental resection followed by reconstruction with tenodesis, tendon transfer, or bridging the defect using allograft or autograft. However, it is unclear which of these treatment options provides the best outcomes for PB tendon tears [12–15].

Reconstruction with semitendinosus allograft has produced satisfactory clinical results and is effective for pain relief and restoration of tendon function [1, 8]. However, there are concerns associated with the use of the allograft, which include its cost and availability, disease transmission, delayed incorporation, and stretching of the graft. The use of hamstring autograft, on the other hand, represents a viable and accessible option that may be biologically superior [15].

The purpose of this study is to describe our surgical technique for the reconstruction of irreparable PB tendon tears using semitendinosus tendon autograft (STA) as an alternative to the allograft and report the short-term results of three cases.

## 2. Materials and Methods

This study reports 3 patients submitted to the reconstruction of the PB tendon tears using STA, from December 2016 to May 2017. Ethical approval was granted by our hospital's HSPM/Ethics Committee, and the study was registered at Clinical Trials National Registry under number 2.880.187. The indication for reconstruction using STA was irreparable tears of the PB tendon. Irreparable tears are defined as the presence of a degenerative tissue associated to longitudinal tears that involves more than 50% of the cross-sectional area of the tendon [9, 10].

### 2.1. Preoperative Planning

**Figure 1:**
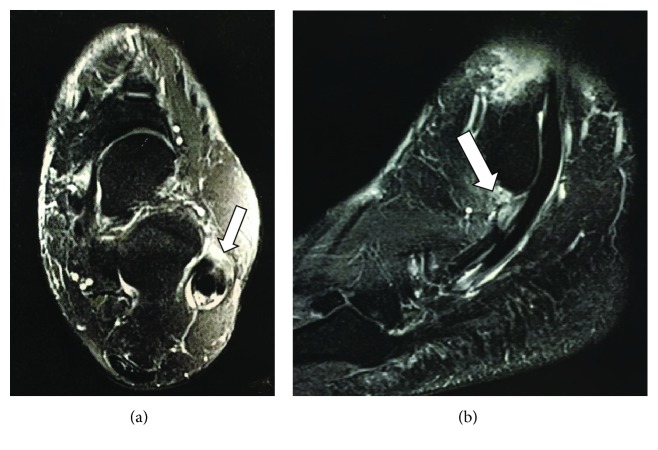
MRI showing PB tendon tears greater than 50% in the (a) axial and (b) sagittal views (white arrows).

A precise clinical evaluation and radiological exams were performed. Clinically, we searched for symptoms and signs of chronic conditions associated to PT injuries such as lateral ankle instability and cavovarus foot. Radiological exams included radiography imaging (RI) and magnetic resonance imaging (MRI). RI included all three views of the ankle as well as Saltzman view to assess hindfoot alignment. Stress X-ray views of the ankle in varus and anterior drawer test were also performed to exclude lateral ankle instability. In the MRI, we evaluated the extent of the PB tendon pathology and possible associated anatomical factors such as a low-lying muscle belly of the PB and peroneus quartus tendon (Figures 1(a) and 1(b)).

### 2.2. Postoperative Follow-up

At 6 months, patients were submitted to an isokinetic evaluation of the strength of both feet in eversion and inversion using an isokinetic dynamometer (CSMI HUMAC Norm, Stoughton, Massachusetts, USA).

## 3. Patients Presentations

In our three patients—(I) a 31-year-old man, (II) a 67-year-old woman, and (III) a 40-year-old man—the mechanism of injury consisted of an ankle sprain. In patient I, the sprain occurred during a soccer match and in patients II and III it occurred while walking in the sidewalk. They went to the outpatient clinic complaining of lateral pain at the hindfoot for 24, 18, and 16 months, respectively. Previous treatments elsewhere were based only on anti-inflammatory drugs, ice, and rest. On a physical exam, there were pain and swelling over the course of the PT. In patients I and II, there were no clinical signs of ankle instability or varus of the hindfoot. In patient III, a bilateral cavovarus deformity was observed. No restriction of the range of motion of the subtalar joint was observed in any of them. Radiographic images were normal. In all MRI images, there were irreparable tears of the PB tendon and anatomical conditions were noted in patients I and II, such as low-lying muscle belly of the PB tendon and a peroneus quartus tendon, respectively. Initially, we conducted a conservative treatment for six months with physiotherapy, rest, analgesics, and ankle stabilizer to restrict inversion-eversion movements, but it was proved unsuccessful.

## 4. Surgical Technique

**Figure 2:**
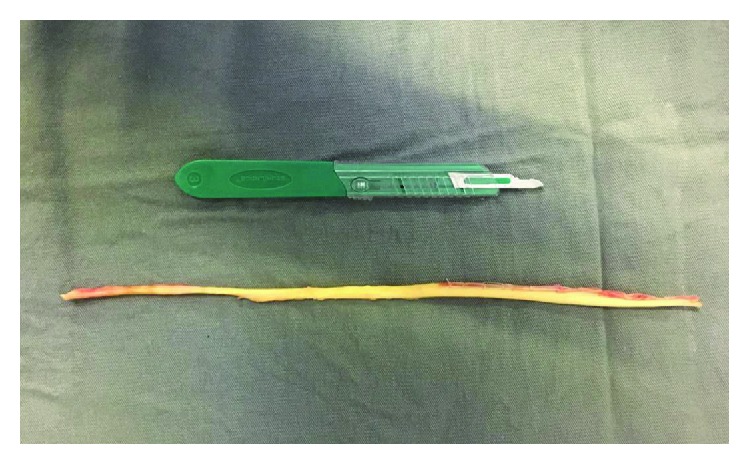
Patient II: the STA was debrided with the removal of its muscle belly, preserving the total length of the tendon.

**Figure 3:**
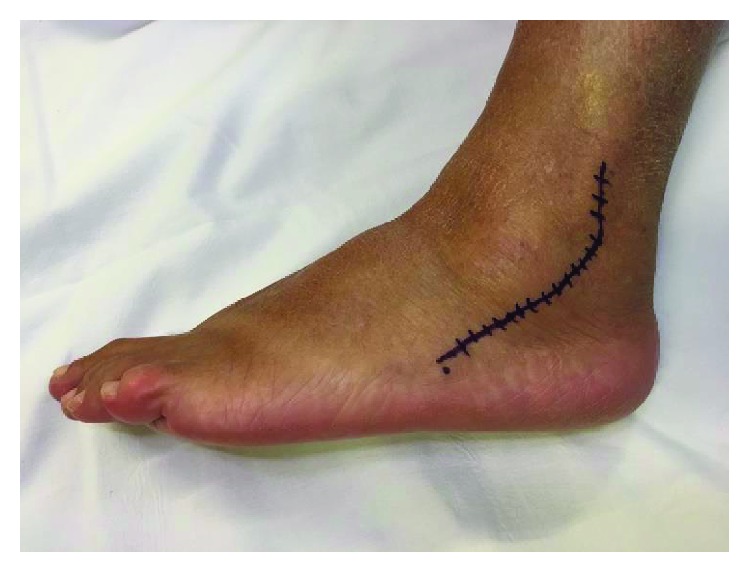
Patient II: the lateral approach is a curved incision starting 3-4 cm above the tip of the lateral malleolus, extending over the course of the PT to the base of the fifth metatarsal.

**Figure 4:**
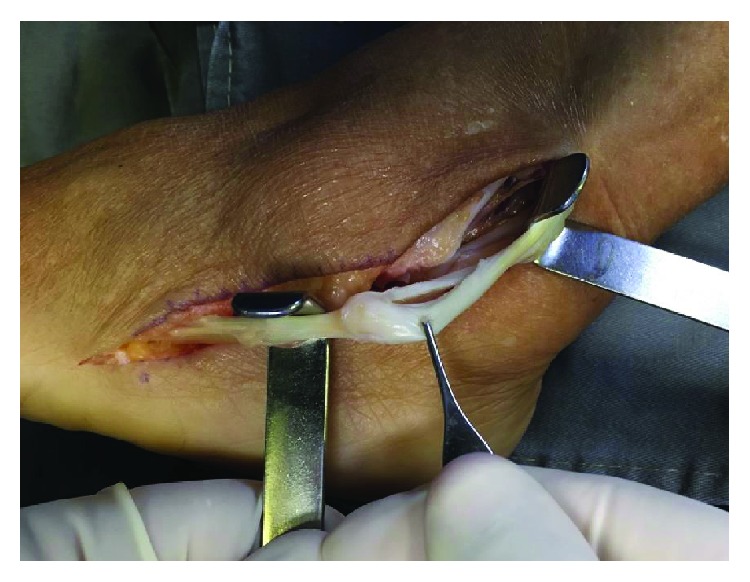
Patient II: extensive alteration of the PB tendon.

**Figure 5:**
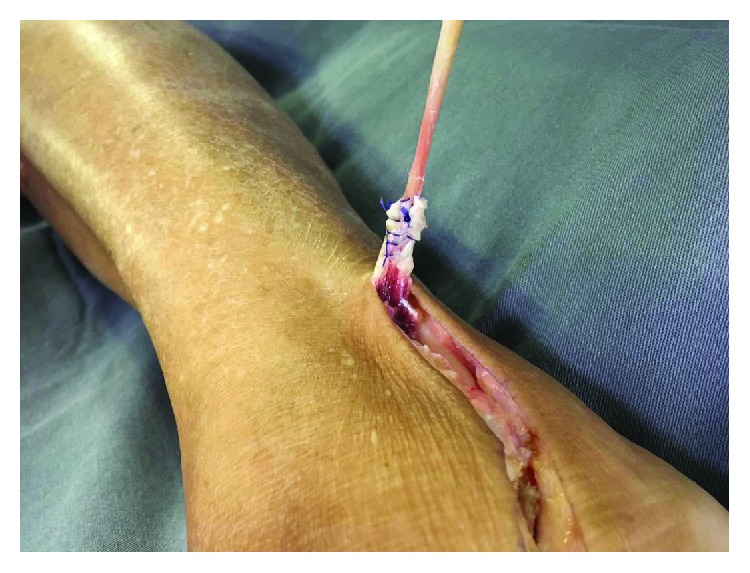
Patient II: the STA is secured to the proximal stump of the PB tendon with a Pulvertaft weave using a 1-0 Vicryl, 3 cm proximal to the tip of the lateral malleolus.

**Figure 6:**
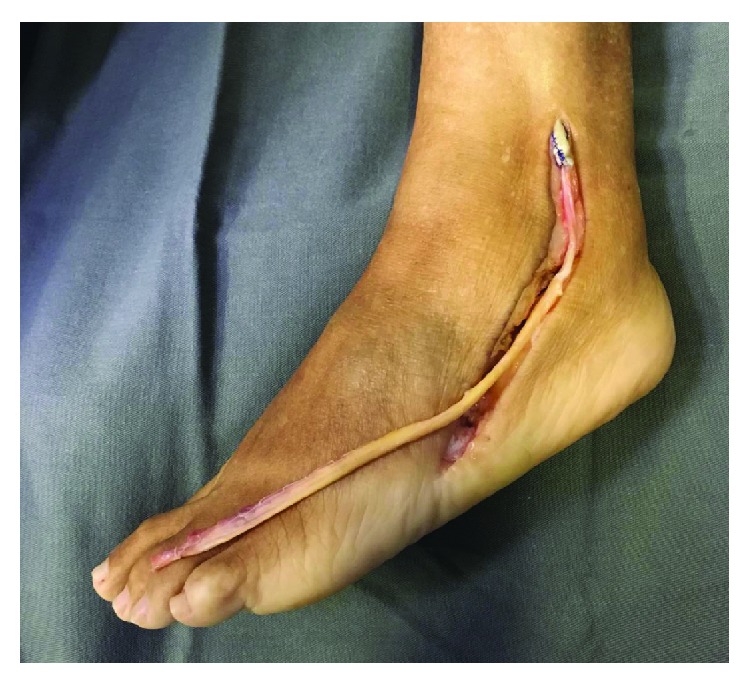
Patient II: aspect of the STA after proximal fixation. We have used the STA in its full length to ensure that there was adequate distal stump length remaining to fix it to the base of the fifth metatarsal.

**Figure 7:**
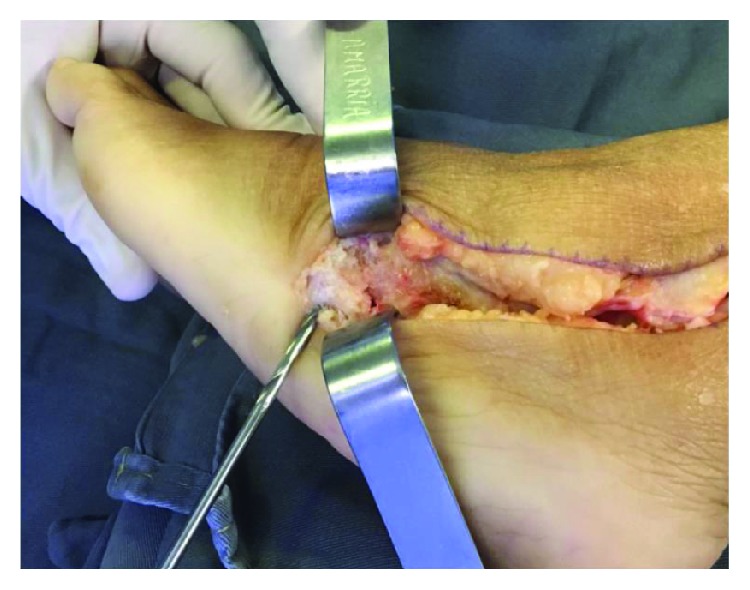
Patient II: the bone tunnel is drilled for a bone-to-tendon fixation with a 3.2 mm drill at the base of the fifth metatarsal perpendicular to the bone, from plantar to dorsal.

**Figure 8:**
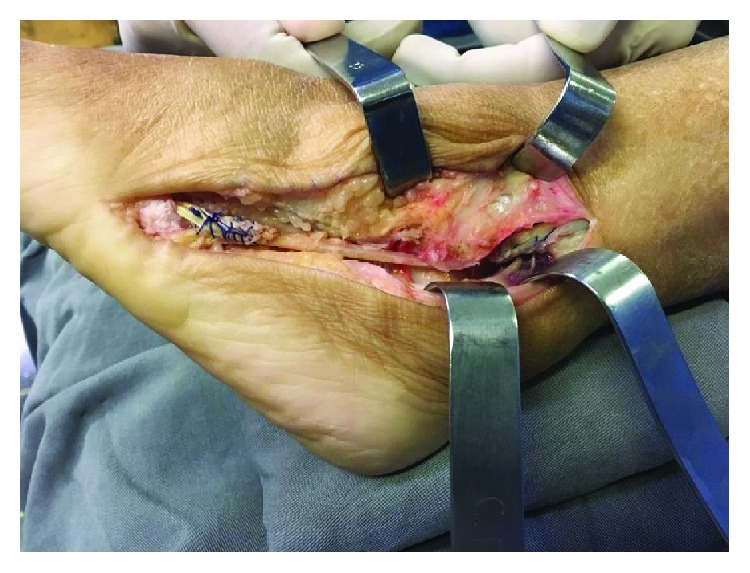
Patient II: bone-to-tendon fixation distal through the bone tunnel after the removal of the remaining portion of the STA.

**Figure 9:**
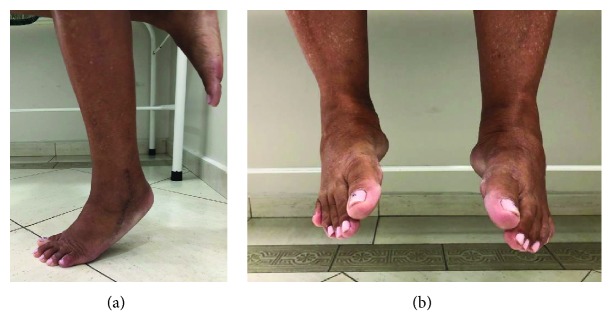
Patient II: (a) single-heel rise test and (b) inversion range of motion at 6 months after surgery.

The illustrative case (patient II) was presented for the demonstration of the surgical technique (Figures 2–9). The surgery was performed with the patient placed in an oblique lateral decubitus under regional anesthesia with a nonsterile thigh tourniquet on a radiolucent operating table. The STA was harvested through a medial longitudinal incision of 3 cm at the region of the proximal lower leg with the hip externally rotated to provide a frontal view of the knee. The graft was prepared by resection of the muscle belly; then its two stumps were tubularized with a 1-0 Vicryl whip stitch. We kept the STA in its full length to ensure that the entire defect was filled after the resection of the unhealthy PB tendon (Figure 2).

A lateral curved incision over the course of the PT was performed along the posterior border of the lateral malleolus, from 3 to 4 cm proximal to the tip of the fibula extending to the fifth metatarsal base (Figure 3). During dissection, care was taken to avoid damage to the sural nerve branches inferior to the lateral malleolus. The peroneal tendon sheath and the superior peroneal retinaculum (SPR) were opened, and PB and peroneal longus (PL) tendons were exposed. Dissection was performed proximally and extended distally to isolate the compromised portion of the tendon (Figure 4). The PB was assessed, and the nonviable portion was resected. The distal stump was debrided and totally removed to prevent local pain due to a bulky suture of the STA to the remaining distal stump, under a thin skin. The STA was sutured to the proximal stump of the native PB tendon using a Pulvertaft suture with a 1-0 Vicryl (Figure 5). The suture was performed 3 cm above the tip of the lateral malleolus to prevent the volume effect of increased pressure within the retromalleolar groove. This distance was based on previous studies which recommend to place peroneal brevis tendon tenodesis to the peroneus longus to avoid pain due to the entrapment of the suture [14]. The distal fixation of the STA was carried out through a bone tunnel at the fifth metatarsal base to provide a bone-to-tendon fixation. Since we kept the full length of the STA, there was an adequate distal stump length remaining to fix it distally (Figure 6). The bone tunnel was drilled with a 3.2 mm drill (Figure 7) perpendicular to the long axis of the fifth metatarsal, from plantar to dorsal. The distal stump was pulled from plantar to dorsal through the tunnel and sutured back to itself with a 1-0 Vicryl (Figure 8). Alternatively, it can be fixed with a biotenodesis screw or an anchor. During the suture, the foot is positioned in neutral inversion/eversion and dorsiflexion/plantarflexion, and the tendon graft was tensioned at 50% of the maximum excursion of the PB muscle belly. The length of the reconstruction was determined at this point. The deep tissues, SPR, and skin were closed in layers. The closure of the SPR was made carefully to prevent PT subluxation. Finally, a sterile dressing and a short leg cast were applied.

### 4.1. Postoperative Rehabilitation

Patients remain non-weight-bearing for two weeks with the cast. At 2 weeks, sutures are removed and they are placed in a walking boot (WB) with full weight-bearing as tolerated. At this period, physical therapy is initiated focusing in the dorsiflexion/plantarflexion range of motion to prevent adhesions in the tendon graft. Inversion-eversion movements are prohibited to prevent stretching of the healing tendon graft and the subsequent development of an elongated tendon with the loss of strength. Patients are instructed to always maintain the WB except for hygiene purposes and dorsiflexion/plantarflexion exercises. The patient who underwent the calcaneal osteotomy followed the same postoperative protocol as the others. At 8 weeks postoperatively, the WB is removed and the patient is transitioned into an ankle-stabilizing orthosis. A physical therapy program is oriented to start inversion-eversion movements and to progressively restore proprioception and strengthening. The ankle-stabilizing orthosis is used progressively less accordingly to the patient rehabilitation.

## 5. Results

In the postoperative period, we noted no skin necrosis, wound dehiscence, autograft rupture, or any associated complications. The three anatomical conditions of the patients associated to the lesions were addressed at the same time, which included a PB low-lying muscle belly resection, a peroneus quartus tendon resection, and a lateral sliding calcaneal osteotomy for a cavovarus deformity.

After 3 months, all patients were pain free, both in the foot and at the donor site, and were able to resume labor activities. After 6 months, all patients could perform a single-heel rise and there was no restriction of the range of motion for inversion (Figures 9(a) and 9(b)). At this point, the isokinetic strength of both feet in eversion and inversion was assessed. The operated feet showed no strength deficit compared to the contralateral side. Eversion strength in patient I was 4%, patient II 4%, and patient III 2% stronger than the contralateral side. At this time, they were allowed to return to sports activities. At a mean follow-up of eighteen months, they were still asymptomatic and fully active.

## 6. Discussion

In 1998, Krause and Brodsky were the first authors to propose a classification system to guide the treatment of irreparable tears of the PT. If less than 50% of the cross-sectional area of the tendon was viable, segmental resection and tenodesis were performed [7, 9]. Although tenodesis is a simple procedure, its clinical outcomes may be unpredictable. Almost two-thirds of the patients report pain on activities and almost 50% of the patients cannot resume full activities. Furthermore, tenodesis sacrifices the functional integrity of the muscle-tendon unit [16].

In 2010, Nunley and Ousema reported for the first time a technique for the management of irreparable tears of the PB tendon using a tendon allograft instead of tenodesis, tendon transfer, or a two-stage procedure. They performed this procedure in 4 patients. A PT allograft was used for the reconstruction of defects greater than 4 cm. The authors presented it as an effective treatment option with satisfactory outcomes. There were no complications associated with this procedure, and all their patients returned to a fully active life [17]. In a retrospective series of 14 patients who underwent a PT (PB and PL tendons) reconstruction with a peroneal or a semitendinosus allograft, Mook et al. showed that all patients returned to their preinjury activity level without pain and yielded satisfactory patient-reported outcomes [8].

Although there is no evidence in which surgical procedure provides the best outcomes, reconstructing the PB tendon with an allograft or autograft seems to be biomechanically superior comparing to tenodesis. Using cadaveric models, Pellegrini et al. compared the effectiveness of allograft reconstruction and tenodesis. They concluded that reconstruction of the PB tendon with allograft substantially restores distal tension when the PT were loaded to 50% and 100% of physiologic load. Tenodesis substantially decreases PB tension under both loads [1].

The literature regarding PB reconstruction with tendon replacement using autograft is scarce. Ellis and Rosenbaum were the first authors to describe a surgical technique for the reconstruction of the diseased PB tendon with STA, but without any clinical results [15]. Although tendon autograft harvest may cause morbidity at the donor site and require a second incision, there are advantages such as tissue compatibility, faster reincorporation, and remodeling rate comparing with the allografts. Recently, Cody et al. published a cohort study of 37 patients with respect to hamstring applications in foot and ankle surgery. Their objective was to evaluate muscle balance and strength of the knee after hamstring autograft harvest with an isokinetic testing. The result was that flexion strength deficit was noted only at the higher degree of flexion of the knee, but without clinical or functional impairment [18]. None of our three patients had residual pain at the donor site, and they were all able to return to their preinjury level of activities after 6 months. Allografts are widely used, but there are concerns including potential disease transmission, risk of immune response, timely incorporation of the graft to the host site, stretching of the graft, and higher costs [19].

To our knowledge, this is the first study to present the surgical technique of reconstruction of the PB tendon using STA with its clinical results. Furthermore, this is the first report to present an objective muscle force measurement during follow-up. Nevertheless, this study has limitations, mainly the small population and the short-term follow-up.

The present study suggests that reconstruction of the PB tendon with STA may be an effective alternative technique to allograft tissues for PB tendon tears. This procedure can decrease pain and restore PB strength without altering foot function. Further studies with larger populations, longer follow-up, and comparisons of PB reconstructions using autograft and allograft are needed to establish the best treatment for these injuries.

## Abbreviations

PT: Peroneal tendon
PB: Peroneus brevis
STA: Semitendinosus tendon autograft
RI: Radiography imaging
MRI: Magnetic resonance imaging
WB: Walking boot.
